# Targeting phosphatidylinositol-3-kinase pathway for the treatment of Philadelphia-negative myeloproliferative neoplasms

**DOI:** 10.1186/s12943-015-0388-z

**Published:** 2015-06-11

**Authors:** Ruchi Pandey, Reuben Kapur

**Affiliations:** Department of Pediatrics, Herman B Wells Center for Pediatric Research, Indiana University School of Medicine, Indianapolis, IN 46202 USA; Department of Microbiology and Immunology, Indiana University School of Medicine, Indianapolis, IN 46202 USA; Department of Medical and Molecular Genetics, Indiana University School of Medicine, Indianapolis, IN 46202 USA; Department of Molecular Biology and Biochemistry, Indiana University School of Medicine, Indianapolis, IN 46202 USA

**Keywords:** PI3K inhibitors, myeloproliferative neoplasms, JAK2, Mastocytosis, Chronic leukemia

## Abstract

Myeloproliferative neoplasms (MPN) are a diverse group of chronic hematological disorders that involve unregulated clonal proliferation of white blood cells. Sevearl of them are associated with mutations in receptor tyrosine kinases or cytokine receptor associated tyrosine kinases rendering them independent of cytokine-mediated regulation. Classically they have been broadly divided into BCR-ABL1 fusion + ve (Ph + ve) or –ve (Ph-ve) MPNs. Identification of BCR-ABL1 tyrosine kinase as a driver of chronic myeloid leukemia (CML) and successful application of small molecule inhibitors of the tyrosine kinases in the clinic have triggered the search for kinase dependent pathways in other Ph-ve MPNs. In the past few years, identification of mutations in JAK2 associated with a majority of MPNs raised the hopes for similar success with specific targeting of JAK2. However, targeting JAK2 kinase activity has met with limited success. Subsequently, mutations in genes other than JAK2 have been identified. These mutations specifically associate with certain MPNs and can drive cytokine independent growth. Therefore, targeting alternate molecules and pathways may be more successful in management of MPNs. Among other pathways, phosphatidylinositol −3 kinase (PI3K) has emerged as a promising target as different cell surface receptor induced signaling pathways converge on the PI3K signaling axis to regulate cell metabolism, growth, proliferation, and survival. Herein, we will review the clinically relevant inhibitors of the PI3K pathway that have been evaluated or hold promise for the treatment of Ph-ve MPNs.

## Introduction

The term ‘myeloproliferative disorders’ has historically been applied to a group of four hematological diseases – 1) polycythemia vera (PV), 2) essential thrombocythemia (ET), 3) primary myelofibrosis (PMF) and 4) chronic myeloid leukemia (CML). This grouping was based on some of the clinical and biological similarities in patients due to clonal proliferation of hematopoietic progenitor cells and excessive production of blood cells of different lineages. With a better understanding of the molecular events underlying these malignancies, the World Health Organization (WHO) has reclassified them on the basis of histology, translocations and mutations in protein tyrosine kinases [1, 2]. These have helped in diagnosis and consequent treatment decisions. Proliferative disorders associated with myeloid cells including the granulocytic, monocytic/macrophage, erythroid, megakaryocytic, and mast cells have been grouped under 5 broad categories: (1) myeloproliferative neoplasms (MPN), (2) myeloid and lymphoid neoplasms associated with eosinophilia and abnormalities of platelet derived growth factor receptor (PDGFR) or fibroblast growth factor receptor (FGFR), (3) myelodysplastic/ myeloproliferative neoplasms (MDS/MPN), (4) myelodysplastic syndrome (MDS) and, (5) Acute myeloid leukemia and related neoplasms. The term ‘myeloproliferative neoplasms’ has replaced the older ‘myeloproliferative disorders’. In addition to the four classical MPNs, PV, ET, PMF and CML (Ph + ve), this group now also includes chronic neutrophilic leukemia (CNL), chronic eosinophilic leukemia not otherwise specified (CEL-NOS), mastocytosis and unclassifiable myeloproliferative neoplasms. For the purpose of this review we will focus on the Ph –ve MPNs as defined under the first category of WHO classification since others have been reviewed elsewhere [3, 4].

As per the estimates for 2008–2010, there were 44–57 and 38–57 PV and ET patients per 100,000 people respectively, while 4–6 PMF patients per 100,000 people in the United States [5]. MF can be primary or develop subsequent to PV/ET. Though its prevalence rate is low, it is the most disabling of the MPNs. No reliable estimates are available for the prevalence rate of mastocytosis, CNL and CEL. The chronic nature of MPNs, absence of a defined therapeutic regimen and potential for transformation to acute myeloid leukemia makes them a challenge. Until a few years back, there was no drug that was approved by Food and Drug Administration (FDA) for the treatment of MPN. Therefore, the treatment options for these patients were mostly empirical [6]. A kinase dependent oncogenic pathway essential for the survival of leukemic cells was identified in chronic myeloid leukemia (CML). The inhibition of the BCR-ABL1 kinase led to significant enhancement in the survival of CML patients [7]. These results along with other studies have contributed to the concept of ‘oncogene addiction’. It is believed that cancer cells are addicted to the oncogene and targeting of the crucial oncogenes/pathways can lead to elimination of cancer cells. However, results from the clinic have demonstrated that cancer cells can acquire additional mutations and activate compensatory pathways that eventually lead to drug resistance and disease relapse [8].

### Genetic basis of MPNs

Discovery of JAK2^V617F^ mutation in a majority of Ph-ve MPN patients and the ability of this mutant protein to recapitulate a MPN phenotype in experimental models has been a turning point in our understanding of classical Ph-ve MPNs [9–11]. It established a genetic basis for the classical Ph-ve MPNs. In addition to becoming an integral part of diagnosis, it has also opened up the field for selective targeting of the kinase pathway in treatment of Ph-ve MPNs. Since then several additional mutations have been identified that specifically associate with different myeloid neoplasms [12, 13]. These include components of the cytokine signaling, regulators of the chromatin structure and RNA splicing in addition to cytogenetic abnormalities with uniparental disomy of chromosome 9p being the most frequent (~35 %) aberration. The driver mutations conferring a clonal proliferative advantage arise in the adult hematopoietic stem cell and early progenitor compartments. Acquisition of a combination of mutations is required for progression from the chronic phase to blast transformation [14, 15]. The number of mutations can range from 1 to 5 or more with or without abnormalities in the karyotype. Such heterogeneity in MPN patients indicates a complex evolutionary mechanism for the disease development. In addition to directing the course of disease progression, the combination of mutations also determines the response to therapy. Therefore, identification of the genetic lesions will be an essential feature of development of effective therapeutic regimens.

These chronic clonal hematopoietic disorders can evolve into acute myeloid leukemia (AML) as seen in 2 – 5 % of PV or ET patients and 15–30 % of the PMF patients. However, the molecular mechanisms that lead to transformation are not well understood. A comparison of mutations in MPNs with those in post-MPN AML shows striking differences in the spectrum of gene sets involved [12]. The genetic lesions in post-MPN AML predominantly involved TP53, TET2, IDH1/2, ASXL1, SRSF2, RUNX1, DNMT3a, CBL along with increase in cytogenetic aberrations that included recurrent deletions (4q, 5q, 6p, 7p, 7q) and uniparental disomy (19q, 22q, 11q) [16]. One of the most interesting features of post-MPN AML was the loss of JAK2^V617F^ mutation in the transformed leukemic blasts [17]. It could be possible that JAK2 mutant clones and leukemic blast clones arise independently in the progenitor compartment or the JAK2 mutations are lost by deletion. An analysis of a cohort of 16 PV patients with leukemic transformation showed that post-PV MF and accelerated phase disease was associated with JAK2 mutated AML while AML with WT JAK2 arose from chronic phase disease possibly as a consequence of genotoxic therapy and damage to non JAK2 mutated HSCs [18]. Mutations in TP53 are rare in the chronic phase but their frequency goes upto 45 % in the leukemic blast phase [19]. These implicate genomic instability and aberrant DNA damage response in the evolution of MPN from a chronic disease to acute leukemia. The precise sequence of mutations and their role in the evolutionary process remain an active area of investigation. Post-MPN AML shares spectra of mutated genes and several cytogenetic features with de novo AML. The increase in frequency of complex karyotype and loss of JAK2 mutations in post-MPN AML is associated with adverse prognosis. The loss of JAK2 mutation is also going to make them resistant to treatment with inhibitors specific for JAK2. Therefore, molecules that target other survival and proliferation regulatory pathways may have better therapeutic value. PI3K pathway has generated much interest in AML and is being investigated in several trials [4, 20]. However, the clinical response has been highly variable and showed limited efficacy possibly due to poor patient selection [21]. A more personalized approach and careful patient selection on the basis of molecular lesions may yield better results. Since MPNs involve clonal expansion of more mature compartments that rely on cytokine responsiveness, the mutations in the components of cytokine signaling module and their effect on the therapeutic outcomes will be discussed in more detail in the next section.

### Pathogenic molecular lesions in cytokine signaling molecules leading to MPNs

**Fig. 1:**
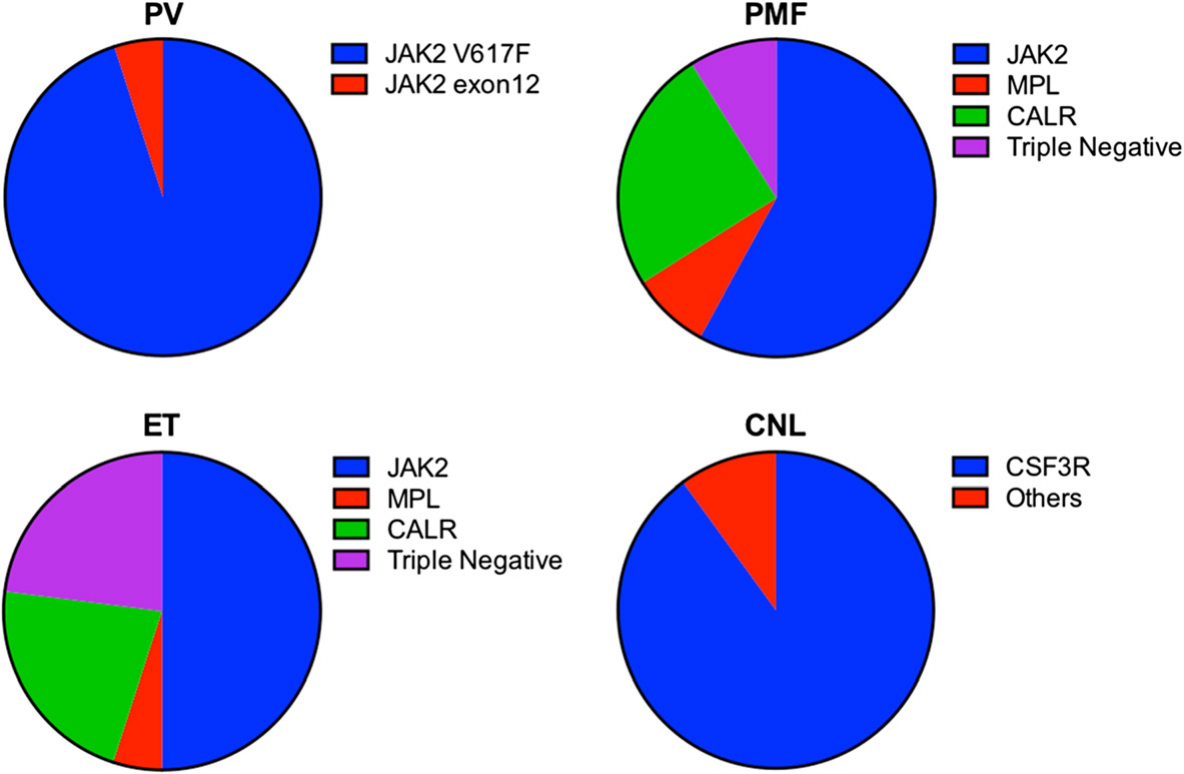
Schematic representation of the distribution of mutations in molecules associated with cytokine signaling in Ph-ve MPNs. The approximate average values are shown

JAK2 is a non-receptor tyrosine kinase that associates with common β chain of IL-3 receptor, gp130 family receptors and IFN-γ receptor and transduces signals downstream of several cytokines [22]. The gain of function V617F mutation leading to constitutive activation of signaling has been detected in over 90 % of patients with PV and in upto 50 % patients with ET and PMF. The rare JAK2^V617F^ negative PV patients carry mutations in the exon 12 of JAK2 that leads to similar constitutive activation of the JAK2 and cytokine independent signaling. Thus, aberrant signaling through the JAK2 pathway accounts for almost all the cases of PV (Fig. 1). However, it is not clear why PV, ET and MF have a different phenotype despite carrying the same genetic mutation. It has been proposed that these differences may be attributable to mutation dose (copy number), differential downstream signaling and presence of additional genetic lesions [23]. In addition to mutations in JAK2, gain of function mutation in thrombopoietin receptor (TPO-R), myeloproliferative leukemia virus oncogene (MPL) at W515 have been reported in ET and PMF. Around 5 % ET and 10 % PMF patients negative for JAK2^V617F^, show presence of JAK2 exon 12 and MPL mutations (Fig. 1) that effectively lead to constitutive activation of the same signaling pathway [24, 25]. Subsequently somatic mutations in calreticulin (CALR), a chaperone protein with known functions in regulating protein folding and cellular calcium metabolism, were detected in 67 % of the ET and 88 % of the PMF patients that were negative for the JAK2 and MPL mutations (Fig. 1) [26]. These mutations were clustered in exon 9 and were absent in PV, AML, CML, MDS and CMML patients or in healthy individuals suggesting that these could serve as specific diagnostic markers for ET and PMF. The mutations involve deletions (53 %), insertions (32 %) and others leading to expression of the protein from an alternative reading frame. The resulting mutant calreticulin protein has a positively charged peptide at the C terminal instead of the negatively charged in the non-mutated protein. Ba/F3 cells expressing the mutated protein acquire IL-3 independent proliferation capacity along with increased phosphorylation of STAT5 though the mechanistic details remain unknown [26]. In addition, loss of function mutations have been reported in negative regulators of cytokine signaling such as adaptor protein LNK (SH2B3) and CBL, a ubiquitin ligase that regulates the proteasomal degradation of some of the cytokine receptors. These mutations will also effectively lead to unregulated constitutive activation of the cytokine signaling pathways. It is interesting to note that mutations in JAK2, MPL and CALR are mostly mutually exclusive and converge on the activation of JAK/STAT signaling pathway. However, in terms of outcome, the median survival time for patients with ET (19.8 years) is longer than for PV (13.5 years) and PMF patients have the shortest median survival (5.9 years) [27]. Stratification of ET and PMF patients on the basis of presence of JAK2, MPL, CALR and triple negative mutations showed no significant survival differences in case of ET but mutational status affected the survival of PMF patients. The triple negative PMF patients had the shortest median survival time followed by JAK2 and MPL mutated, respectively. The longest survival times were observed in CALR mutated patients [27]. Surprisingly, this survival advantage in CALR mutated patients was restricted to those carrying the deletion mutations in CALR and in presence of insertion mutations the median survival times were similar to JAK2 mutated patients though both type of mutations result in change in charge and loss of ER retention motif at the C-terminus of the protein [28]. Mutations in chromatin regulators are frequent events in these MPNs and patients carrying ASXL1, IDH, EZH2 or SRFS2 are at higher risk. Presence of ASXL1 mutation along with CALR mutation was associated with reduction in median survival times but the worst prognosis was observed in patients having mutation in ASXL1 and non-mutated CALR [29]. These results further highlight the importance of defining the different genetic lesions and consequent signaling mechanisms for developing effective therapeutic regimen in MPNs.

**Fig. 2:**
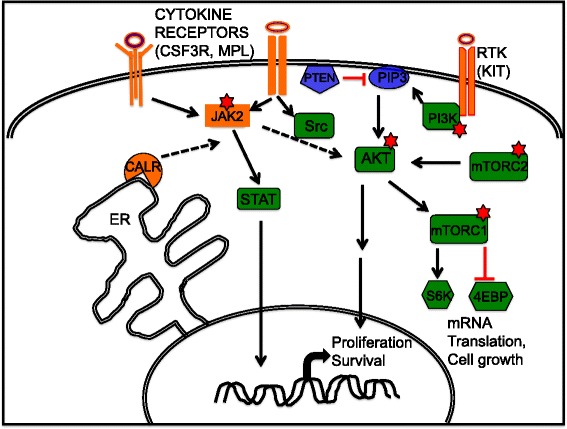
Schematic showing the PI3K pathway and the targets for therapy. Red stars indicate the molecules that are being specifically targeted by small molecule inhibitors as single agents or in combination

Mastocytosis, the newly added subgroup to Ph-ve MPNs, involves clonal proliferation of mast cells and their accumulation in different organs. Mature mast cells express tyrosine kinase KIT, receptor for stem cell factor and activating mutations in KIT are present in mastocytosis and AML patients. Mutations at D816 are present in majority of the adult systemic mastocytosis (SM) patients and are also frequently associated with pediatric cutaneous mastocytosis [30–32]. Additional mutations in KIT, F522C, M541L, V560G, D820G and E839K, have also been reported in mastocytosis patients. Similar to JAK2 mutations, the gain of function mutations in KIT leads to constitutive activation of downstream signaling pathways and cytokine independent proliferation of the mutant cells. Tyrosine at position 719 within the cytoplasmic domain of mutant KIT plays an important role in the growth factor independent activation of STAT5 and PI3K/AKT/mTOR pathways [33]. Activation of the PI3K pathway contributes to cell survival through activation of Rho GTPase and Rho-associated coiled coil-containing protein kinases (ROCK) and is essential for the transformation by KIT D816V [34]. Some of the KIT mutations are sensitive to inhibition by tyrosine kinase inhibitor, Imatinib but D816V mutation present in a majority of systemic mastocytosis patients is resistant to Imatinib [35, 36]. Dasatinib, a second-generation multi-kinase inhibitor, is able to inhibit KIT mutations that are resistant to Imanitib including D816V but it has met with little success in the clinic [37, 38]. Thus, despite 2 decades of research to identify inhibitors of KIT, a cure for SM, an aggressive malignancy is still not available. This has forced the researchers to evaluate other downstream mediators of mutant KIT signaling such as SHP2 phosphatase and p21 activated kinase (PAK) as potential druggable targets [39–41].

A relatively rare, chronic neutrophilic leukemia (CNL) characterized by elevated neutrophil counts is also included as a myeloproliferative neoplasm in the WHO 2008 classification. A search for molecular basis of CNL pathogenesis showed that deregulated signaling through mutations in cytokine receptor might be pivotal in development of CNL also. Truncation and membrane proximal mutations in colony-stimulating factor 3 receptor (CSF3R), a transmembrane receptor, that lead to cytokine independent proliferation appear to be specifically associated with CNL [27–29]. CSF3R regulates survival, proliferation and differentiation of granulocytes in response to granulocyte colony stimulating factor (G-CSF). T618I mutation in the membrane proximal region with or without additional mutations that truncate the cytoplasmic domain of CSF3R was observed in more than 80 % patients diagnosed with CNL as per the WHO criteria [42–44]. Recapitulation of a fatal neutrophilic leukemia phenotype upon transplantation of CSF3R T618I mutation expressing hematopoietic cells further established a causal role for CSF3R mutation in development of CNL [45]. Mutations in CSF3R can therefore be a potential diagnostic marker for CNL. The membrane proximal point mutations vs truncation mutations in CSF3R may also have prognostic and clinical relevance. The membrane proximal mutations (T615A and T618I) in CSF3R were more potent in acquisition of cytokine independent growth and colony formation as compared to the truncation mutations due to activation of different signaling modules. The membrane proximal mutations activate the JAK2/STAT3 signaling axis and are sensitive to JAK inhibitor [42, 45] but insensitive to tyrosine kinase inhibitor Dasatinib. While the truncation mutations activate SRC family kinase, FGR/tyrosine kinase non-receptor 2 signaling axis and are sensitive to dasatinib but resistant to JAK inhibitors [42]. In addition presence of mutations in SETBP1, encoding a nuclear protein that binds to DNA replication associated SET oncogene, determines the prognosis and response to therapy. Mutations clustered in the D868 to D874 region of SETBP1 were found in 30 - 40 % of the CNL patients and was associated with shorter life span. Interestingly, presence of SETBP1 mutation in addition to CSF3R T618I conferred resistance to not only conventional hydroxyurea therapy but also to JAK2 inhibitor, ruxolitinib [46]. Thus, JAK2 inhibition may be effective when only CSF3R T618I mutation is present but is likely to be ineffective in presence of CSF3R truncation or additional mutations such as SETBP1. Though it is not known which pathways are activated by SETBP1 that contribute to the resistance of double mutant cells. Since this data is based on a study with a single patient, further studies in with larger sample size will be required to establish the functional role of SETBP1 mutations.

Chronic eosinophilic leukemia, not otherwise specified (CEL-NOS) is another rare disease characterized by clonal expansion of eosinophils that has been included as a myeloproliferative neoplasm in the current classification system by WHO. CEL-NOS is highly aggressive with poor prognosis and high mortality. In a cohort of 10 CEL-NOS patients the median survival from the time of diagnosis was only 22.2 months [47]. 50 % of the patients had acute transformation with median time of 20 months, and died with median survival of 2 months (range 1.0- 6.1) after acute transformation [47]. Since it is a rare disease, the small sample size makes it difficult to make conclusions about the molecular lesions that contribute to the development of this disease. In a recent study, Iurlo et al., [48] identified gain of function M541L mutation in KIT in 4 out of 5 CEL-NOS patients with non-mutated JAK2, BCR-ABL1, FIP1L1/PDGFRα and TEL/PDGFRβ. These patients showed complete hematological remission upon treatment with low dose tyrosine kinase inhibitor (TKI) Imanitib, and were disease free upto 6 years after treatment with only one relapse occurring after 4 years [48]. These results are in stark contrast to poor prognosis and high mortality in CEL-NOS patients with conventional treatments [47]. Therefore, though presence of KIT^M541L^ may not be a molecular marker for CEL-NOS as it has also been observed in pediatric mastocytosis [32] but identification of the mutational status can make a significant difference in the treatment decisions and outcome of this disease.

### Inhibition of JAK signaling in treatment of MPN

Aberrant regulation of cytokine dependent signaling pathways appears to be the unifying molecular feature in all the Ph-ve MPNs. Acquisition of the mutations discussed above lead to enhanced cytokine sensitivity and clonal proliferation of lineage committed cells. In the absence of knowledge of the molecular mechanisms, the treatment options were limited to phlebotomy, hydroxyurea, interferons, and immunomodulatory agents to provide symptomatic relief to the patients [6]. JAK2 has been the target of choice since JAK2^V617F^ was the first and is the most frequent mutation associated with classical Ph- ve MPNs. Ruxolitinib (INC424/ INCB018424/ Jakafi), a specific inhibitor of JAK kinases, was the first inhibitor to be approved by FDA for the treatment of high and intermediate risk MF and PV. There was a marked and durable decrease in spleen size and disease associated pathologies upon treatment with Ruxolitinib as compared to the standard available therapy leading to improvements in the quality of life [49, 50]. Success with Ruxolitinib raised the hopes but it was not curative. Additionally, treatment with Ruxolitinib was associated with thrombocytopenia and anemia in a significant number of MF patients undergoing the phase III trial. The beneficial effects of Ruxolitinib though encouraging, turned out to be limited in scope. [51, 52]. Partial success with Ruxolitinib has spurred the search for other JAK kinase inhibitors with improved efficacy and reduced toxicity. Pre clinical and clinical data with Fedratinib (TG101348/SAR302503), the second JAK kinase inhibitor to have entered phase III clinical trial, were promising but further development had to be discontinued following Wernicke’s encephalopathy in these patients [53]. Similarly, clinical trials had to be terminated and further development discontinued due to severe pleiotropic neurological adverse effects in patients treated with JAK inhibitors, AZD1480 (clinical trial NCT01219543) or XL019 [54, 55]. None of the JAK2 inhibitors are specific to the mutated protein and in addition to JAK2 they can also inhibit JAK1 activity thereby leading to non-specific effects and toxicity. While new molecules continue to be evaluated for targeting the JAK/STAT pathway, a combinatorial approach is also being tested to reduce the toxicity and increase the efficacy [56, 57]. Some of these combinations are designed to target the side effects of Ruxolitinib therapy such as anemia, MPN associated fibrosis, or the histone acetylation and DNA methylation which is frequently deregulated by the mutations in the regulators of the epigenome (Table 1). However, not all MPNs have mutations in JAK2 or activate the JAK/STAT signaling and are therefore, not likely to respond to JAK2 inhibition therapy. Some of the mutations, such as those in c-KIT can directly activate PI3K and other signaling pathways. In addition, very little is known about the mechanisms that contribute to enhanced proliferation and cytokine responsiveness in the triple negative group in PMF patients. PI3K/AKT/mTOR is a central signaling module and several other pathways converge on it to regulate cell survival and proliferation. It is also constitutively activated in MPNs and targeting it either alone or in combination has yielded encouraging results. Since the PI3K/AKT/mTOR signaling axis is deregulated in various other cancers, significant efforts have been channeled to identify small molecular inhibitors of this pathway. These molecules are already in various stages of pre clinical and clinical evaluations. Therefore, these inhibitors of PI3K pathway can become rapidly available to MPN patients.

**Table 1:** Ongoing clinical evaluations of ruxolitinib in combination with other compounds for treatment of MPNs

Compounds	Target/mechanism	National Clinical Trial Identifier	Phase	Condition	Status
Decitabine	Anti-metabolite chemotherapeutic, DNA demethylation	NCT02076191	I/II	Accelerated or blast phase MPN	Recruiting
Azacitidine (Vidaza)	Chemical analogue of nucleoside cytidine, antimetabloite chemotherapeutic	NCT01787487	III	MF, MDS/MPN	Recruiting
		NCT02257138	I/II	AML, post MPN-AML	Active, not recruiting
Danazol	Synthetic steroid derived from ethisterone, used in endometriosis treatment	NCT01732445	II	Anemia in PMF, post ET/ PV MF	Recruitment suspended for interim data to mature
Pracinostat (SB939)	Hydroxamic acid based histone deacetylatase inhibitor of class 1/2/4 HDACs	NCT02267278	II	MF	Active, not recruiting
Panobinostat (LBH589)	Hydroxamic acid based non selective HDAC inhibitor	NCT01693601	I/II	MF	Recruiting
		NCT01433445	I	PMF, post ET/ PV MF	Active, not recruiting
Pomalidomide (CC-4047-MM-002)	Derivative of thalidomide, inhibits angiogenesis, immunomodulator	NCT01644110	I/II	PMF, post ET/PV MF	Recruiting
Lenalidomide	Anti-angiogenic, immunomodulator	NCT01375140	II	MF	Active, not recruiting
Simtuzumab (GS-6624)	Humanized monoclonal antibody, binds to LOXL2 and inhibits fibrosis	NCT01369498	II	PMF, post ET/PV MF	Completed
PRM-151	Recombinant pentraxin-2 protein (acute immunological responses) inhibits fibrosis	NCT01981850	II	PMF, post ET/PV MF	Recruiting
BKM120	PI3K inhibitor	NCT01730248	I	PMF, post ET/PV MF	Recruiting
LDE225	Investigational inhibitor of Smoothened (SMO), a regulator of hedgehog signaling pathway	NCT01787552	Ib/II	PMF, ET, MPD	Recruiting

The clinical trials registry and database [53] was searched for ongoing clinical trials of Ruxolitinib (Jakafi/ INCB18424) in combination with other therapeutics and studies enlisting MPN patients were tabulated. The information presented is current as of 24^th^ January 2015

### Phosphatidylinositol 3 Kinase pathway in MPN

There are three classes of PI3K and the focus will be on class IA since it plays a central role in normal development as well as in the pathogenesis of different cancers. Phosphatidylinositol 3 kinase pathway is activated at the cell surface and integrates signals from cytokines, growth factors and environmental cues and transmits them through AKT and mTOR to effector molecules that control protein synthesis, growth, survival and proliferation [58, 59]. The active PI3K enzyme consists of a heterodimer of a catalytic subunit and a regulatory subunit. The catalytic subunits p110α, p110β, and p110δ are encoded by Pik3ca, Pik3cb, and Pik3cd, respectively while Pik3r1, Pik3r2, and Pik3r3 encode the class IA PI3K regulatory subunits p85α, p85β and p55γ, respectively. Alternate codon usage from Pi3r1 generates two additional regulatory units, p55α and p50α. There is no specificity with regard to association between regulatory and catalytic subunits. Any regulatory subunit can associate with any of the catalytic subunits present. Cell type specificity in PI3K activity is achieved through cell lineage specific expression of the different isoforms. Class 1 PI3K utilize the lipid substrate, phosphatidylionsitol-45, biphsophate (PIP2) and catalyze the phosphorylation at 3-position in the ring to generate the active second messenger, phosphatidylinositol3,4,5- triphoshpate (PIP3). In unstimulated cells, the regulatory subunit binds to the catalytic subunit and inhibits the catalytic activity. Upon stimulation, the regulatory subunit is recruited to the phospho-tyrosine proteins present at the cell membrane and the catalytic unit catalyzes the formation of PIP3 that in turn acts as a tethering moiety for AKT. Upon recruitment to the membrane, AKT is phosphorylated and in turn activates mammalian target of rapamycin (mTOR), a serine threonine kinase and other proteins involved in cell survival and proliferation (Fig. 2). The signaling is attenuated by the lipid phosphatases, PTEN and SHIP that dephosphorylate and convert PIP3 back to PIP2 and the inhibitory feedback circuitry [60, 61].

Hyperactivation of the PI3K signaling module has been observed in a large number of solid cancers and hematological malignancies. This may either be due to oncogenic activation of receptor tyrosine kinases and/or also due to increased expression, copy number gain, amplifications or mutations in Pik3ca, Pik3r1, AKT, TSC1/2 and PTEN [62, 63]. In MPN patient bone marrow biopsy samples with JAK2^V617F^ or KIT^D816V^ mutation, increase in phosphorylation of AKT indicates constitutive activation of the PI3K pathway [64]. PI3K regulatory subunits p85α and p85β appear to have different roles in transformation of myeloid cells by mutant KIT. While genetic deletion of p85α abrogated the cytokine independent growth and leukemogenic activity of KIT^D814V^, deletion of p85β had no effect on the growth factor independent proliferation [65, 66]. Similarly, consistent with the selective expression of the p110δ catalytic subunit in the leucocytes, specific inactivation of p110δ but not that of p110α inhibited the hypersensitivity to GM-CSF due to presence of activating mutation in Shp2 phosphatase and subsequent activation of the PI3K signaling module [67]. In addition to contributing to pathogenesis of neoplasms, constitutive activation of PI3K pathway also contributes to resistance to tyrosine kinase inhibitors (TKI). This development of resistance is not due induced mutations in BCR-ABL1 [68]. The epigenetic silencing of PTEN in response to therapy and consequent activation of the PI3K/AKT along with other pro-survival pathways possibly mediates drug resistance [69]. Given the crucial role of PI3K signaling module there is intense interest in finding specific drugs and more than 40 molecules by different pharmaceutical companies are in different stages of clinical evaluations (Table 2).

**Table 2:** Inhibitors of the PI3K/AKT/mTOR pathway in the clinic

Compound	Target specificity	Availability	Mechanism	Phase	Cancer
Afuresertib (GSK2110183)	AKT 1/2/3	Oral	ATP competitive	I, II	MM, CLL, Solid cancers
BAY1125976	AKT 1/2	Oral	Allosteric, Non-ATP competitive	I	Advanced solid tumors
RX-0201 (Archexin)	AKT 1	IV	Antisense oligodeoxymucleotide	I/II, II	Pancreatic, Renal
ARQ 092	Pan AKT, selectivity for AKT1	Oral	ATP competitive	I	Solid tumors, recurrent Lymphoma
GDC-0068	AKT 1/2/3	Oral	Non-ATP competitive	I, II	Solid cancers
Uprosertib (GSK2141795)	AKT 1/2/3	Oral	ATP competitive	II	Solid cancers, AML, MM
MK2206	Pan AKT, selectivity for AKT1	Oral	Allosteric, Non-ATP competitive	II	Solid tumors, lymphoma
SR13668	AKT	Oral	Blocks AKT phosphorylation	I	Healthy volunteers (completed)
LY2780301	AKT/p70S6K	Oral	ATP competitive	I/II	Advanced solid tumors, non-hogdkin’s lymphoma
AZD5363	AKT, 1/2/3 P70S6K/PKA	Oral	ATP competitive	I/II	Solid cancers
ONC201 (TIC10)	AKT/ERK	Oral	Indirect	I/II	Advanced solid tumors
BKM120 (buparlisib)	PI3K	Oral	ATP competitive	II, III	Advanced cancers
ZSTK474	PI3K	Oral	S-triazine derivative, ATP-competitive	I/II	Advanced solid tumors
Copanlisib (BAY 80–6946)	PI3K	IV	Imidazolinoquinazoline derivative	I. II	Advanced solid tumors, NHL
PX-866	PI3K	Oral	Wortmannin analogue, irreversibly binds catalytic site	I/II	Solid tumors
XL147 (SAR245408)	PI3K (MEK/ERK)	Oral	ATP competitive	I	Advanced solid tumors
BYL719	PI3K alpha	Oral	2-aminothiazole derivative	I/II	Advanced solid tumors
INK1117 (MLN1117)	PI3K alpha	Oral		I	Metastatic solid tumors
AZD8186	PI3K beta	Oral		I	CRPC, sqNSCLC, TNBC, PTEN deficient advanced cancers
GSK2636771	PI3K beta	Oral	substituted benzimidazole	I/II	CRPC, PTEN deficient solid tumors
Idelalisib (Zydelig, CAL-101, GS-1101)	PI3K delta	Oral	Quinazoline class	III	NHL, FDA approved for relapsed CLL, SLL, FL
INCB040093	PI3K delta	Oral		I	B cell malignancies
INCB050465	PI3K delta			I	Relaposed/ refractory B cell malignancies
BAY1082439	PI3K alpha/beta	Oral		I	Advanced solid tumors
GDC-0941 bismesylate	PI3K alpha/delta	Oral	ATP competitive	I/II	TNBC, NSCLC
AZD8835	PI3K alpha/delta	Oral		I	Advanced solid tumors
GS-9820 (CAL-120)	PI3K beta/delta	Oral		Ib	Lymphoid malignancies
RP6530	PI3K gamma/delta	Oral		I	Hematological malignancies
IPI-145	PI3K gamma/delta	Oral	ATP competitive	I, III	Hematological malignancies, follicular lymphoma
DCBCI0901	PI3K/mTORC1/2	IV		I	Advanced solid tumors
P7170	PI3K/mTOR/DNA-PK/ALK-1	Oral		I	Advanced refractory tumors
VS-5584	PI3K/mTOR	Oral		I	Non hematological cancers, metastatic cancers, Lymphoma
DS-7423	PI3K/mTOR	Oral		I	Solid tumors, completed
PF-05212384	PI3K/mTOR	IV		I	Advanced solid tumors
BEZ235	PI3K/mTOR	Oral	Imidazo (4,5) quinoline derivative, ATP competitive	I/II	Advanced solid tumors
XL765 (SAR245409)	PI3K/mTOR	Oral		I/II	Advanced solid tumors
BGT226	PI3K/mTOR	Oral		I/II	Advanced solid cancers, completed
GDC-0980	PI3K/mTOR			I/II	NHL, EC, Solid tumors
GSK2126458	PI3K/mTOR	Oral	Pyridylsulfonamide derivative	I	Refractory solid tumors
PWT33597 mesylate (VCD-597)	PI3K alpha/mTOR	Oral	Derived from pan-Class I PI3-kinase ATP competitive inhibitor ZSTK474	I	Advanced malignancies
MLN0128 (INK128)	TORC1/2	Oral	ATP competitive	I,II	Advanced solid and hematological malignancies
OSI-027	TORC1/2	Oral	4,5,7-trisubstituted imidazo[5,1-*f*] triazine	I	Solid tumor, lymphoma
AZD8055	TORC1/2	Oral	ATP competitive, inhibits kinase activity	I	Advanced cancers
AZD2014	TORC1/2	Oral	ATP competitive	I/II	Advanced solid cancers
CC-223	TORC1/2	Oral	ATP competitive	I/II	MM, DLBCL, NHL, solid cancers
ME-344 (NV-128	mTOR	IV	Isoflavone derivative	I	Solid tumors
ABTL0812	TORC1/2, DHFR	Oral	Lipid analogue	I	Advanced tumors
CUDC-907	PI3K/HDAC	Oral		I	Advanced solid tumors, MM

The drug dictionary maintained by National Cancer Institute (http://www.cancer.gov/publications/dictionaries/cancer-drug) was mined for inhibitors of the PI3K signaling module and information on their status in clinical trials was obtained from the clinical trial registry database [53]. Abbreviations: MM- multiple myeloma, CLL – chronic lymphocytic leukemia, AML – acute myeloid leukemia, NHL – Non Hodgkin’s lymphoma, TNBC – triple negative breast cancer, CRPC – castration resistant prostate cancer, NSCLC - Non-small cell lung carcinoma, SLL - small lymphocytic lymphoma, FL- follicular B-cell non-Hodgkin lymphoma, DLBCL - Diffuse large B-cell lymphoma, IV - intravenous

### Targeting the PI3K Pathway

The PI3K signaling module has emerged as one of the most attractive target in cancer therapeutics due to its central role in integrating signals from different receptor kinases to regulate cell survival and proliferation. Significant efforts have been devoted to identify small molecule inhibitors that can block the PI3K pathway at one or more of the signaling nodes (Table 2). Accordingly, they can be broadly classified as inhibitors of (1) PI3K (2) AKT and (3) mTOR (Fig. 2). Additionally some inhibitors can target the PI3K pathway at more than one node and they are grouped as dual-specificity inhibitors. These molecules are in various stages of pre-clinical and clinical evaluation either as single agents or in combination with other therapeutics in advanced solid tumors and hematological malignancies. Though some of these molecules hold promise, so far the success has been limited. Clinical trials with GSK1059615, a PI3K α inhibitor (NCT00695448), GSK690693, inhibitor of AKT (NCT00493818) and AZD2014, mTORC1/2 inhibitor (NCT00493818) had to be terminated due to limited efficacy or drug related toxicities [70]. While further development of GSK1059615 and GSK690693 has been stopped, AZD2014 is being evaluated in several clinical trials in combination with other agents (Table 2). Some of the PI3K pathway inhibitors have also been evaluated in models of MPN and have entered the clinic as discussed below. As the safety and efficacy of these small molecules is established in the early clinical trials with solid cancers and lymphomas, clinicians will have more options to choose from while designing the therapeutic strategies for MPN patients based on the mutational status and molecular markers. Interestingly, majority of the small molecule inhibitors of the PI3K signaling module have oral bioavailability (Table 2) which is better suited for the management of chronic diseases like MPN and ensuring patient compliance.

#### Inhibitors of PI3K

The inhibitors of the class IA PI3K can be further subdivided into pan PI3K inhibitors and isoform specific inhibitors. The first generation pan Class I PI3K inhibitors, Wortmanin and LY294002 had potent activity in vitro model systems but they did not make it to the clinic due to severe toxicities observed in vivo. PX-866, a synthetic derivative of wortmannin and irreversible pan PI3K inhibitor, was found to be safe and well tolerated in patients who had already undergone 3 or more therapies for advanced solid cancers [71]. Interestingly, PX-866 also inhibited TGF-α/EGFR induced lung fibrosis in preclinical models when used as a single agent or in combination with MEK inhibitor [72, 73]. These results raise the hope that inhibition of the PI3K pathway by PX-866 may be effective in managing PMF and post ET/PV-MF. BKM120 is another promising pan class I PI3K inhibitor that has reached phase III in clinical trials with advanced solid tumors (Table 2). In addition to inhibiting the tumor cell growth, BKM120 also blocked the protective signals from the microenvironment in preclinical models of T-ALL and CLL [74, 75]. BKM120 is currently being investigated in the clinic in combination with ruxolitinib for treatment of MF (Table 1). However, it remains to be seen if any of these PI3K inhibitors will be successfully applied for the management of MPNs.

While promising leads have been obtained with pan class I PI3K inhibitors, efforts have also been directed to develop isoform specific inhibitors to limit systemic toxicities (Table 2). While p110α and β isoforms have a more ubiquitous tissue distribution, p110δ has a more restricted expression in the hematopoietic system. Therefore, inhibitors directed at p110δ catalytic subunit are being preferentially evaluated in hematological malignancies (Table 2). Idelalisib (also known as Zydelig, CAL-101, GS-1101) became the first in this class of inhibitors to get FDA approval for use in relapsed and refractory CLL, follicular B-cell non-Hodgkin lymphoma and relapsed small lymphocytic lymphoma. In the clinic, Idelalisib is efficacious as a single agent as well as in combination with rituximab in CLL patients who had treatment refractory disease or were unsuitable for standard chemotherapy [76, 77]. In pre-clinical evaluations, a PI3K α/δ inhibitor, GDC-0941 inhibited the proliferation driven by mutant KIT in 32D cells [33] while Idelalisib and another PI3K δ specific inhibitor, GS-9820 (Table 2) inhibited the GM-CSF induced proliferation of bone marrow cells expressing the constitutively active Shp2 phosphatase mutant with concomitant reduction in AKT phosphorylation [67]. These results indicate that isoform specific inhibition of p110 catalytic subunit may also benefit MPN patients. Idelalisib is particularly interesting as in addition to induction of apoptosis in the leukemic cells, it antagonizes the protective effect of bone marrow stroma on CLL cells by blocking the expression of BCR induced lymphocyte cytosolic protein 1 (LCP1). It also interferes with cytoskeleton rearrangement and migration thereby leading to CLL cell de-adhesion and apoptosis [78, 79]. IPI-145, inhibitor of p110 δ and γ isoforms has also been found to be safe and efficacious in phase I trial. Notably, IPI-145 also antagonized extracellular survival signals and sensitized ibrutinib resistant cells with BTK^C481S^ mutation [80]. IPI-145 also exerts anti-inflammatory activity through modulation of chemokine secretion, neutrophil migration and activation of basophils [81]. It is interesting to note that some of these isoform specific inhibitors are not only toxic to the transformed cells but can also counter the protective effects of the microenvironment and reduce inflammation, two of the critical contributors to leukemic transformation and resistance to therapeutics in vivo. Taken together these results provide a strong rationale for evaluation of PI3K inhibitors in MPN models but the success is likely to be dependent upon patient selection on the basis of genetic lesions.

#### Inhibitors of mTOR

Mammalian target of rapamycin (mTOR), a serine threonine kinase with homology to the PI3K, is present as part of two different complexes – mTORC1 and mTORC2 (Fig. 2). mTORC1 includes raptor, mLST8, PRAS40 and DEPTOR and is mainly involved in regulating cell growth and protein synthesis through its downstream effectors, S6 kinase and 4E-Binding Protein 1 (4EBP1). On the other hand, mTORC2 complex includes rictor, mLST8, DEPTOR, mSin1 and protor. mTORC2 plays a role in survival signaling through phosphorylation of AKT^S473^ and serum and glucocorticoid-inducible kinase (SGK). Rapamycin was the first allosteric inhibitor of the mTOR complex and has led to development of next generation inhibitors referred to as rapalogs that include everolimus and temsirolimus [82]. These primarily inhibit mTORC1 by interacting with FK binding protein 12 (FKB12) and have little or no effect on mTORC2. Differential expression of mTORC1 and mTORC2 in immature and neoplastic mast cells versus the terminally differentiated mast cells may provide a therapeutic window for selectively targeting the mTORC2 [83]. It may be possible to inhibit the proliferation of only the neoplastic cells while leaving the functionality of terminally differentiated mast cells intact. Rapamycin was reported to selectively inhibit phosphorylation of 4E-BP1 and induce apoptosis in mast cells with D814V mutation [84]. These results suggested that targeting mTOR might be successful in treatment of systemic mastocytosis. However, no clinical benefit was observed in a phase II clinical trail with everolimus (RAD001) in a cohort of 10 patients with either indolent or aggressive systematic mastocytosis [85]. However it is worth noting that 6 of these 10 patients had been treated with other agents previously and had possibly activated compensatory mechanisms that might have made them resistant to therapy. More promising results were observed in another phase I/ II clinical trial with everolimus in a cohort of 39 patients with primary and post-PV/post-ET MF even though in this study too over 80 % of the patients had been unresponsive to previous treatment with at least one conventional therapeutic regimen of either cytotoxic drugs, interferon therapy, erythropoiesis stimulation, or immunomodulatory agents [86]. 44 % response rate was observed for splenomegaly while complete resolution of constitutional symptoms and pruritus was reported in 69 % and 80 % of the patients, respectively with modest hematological and non-hematological toxicities. Patient response showed strong correlation with reduction in p70S6K phosphorylation in responders while no significant differences were observed in the plasma proteins or inflammatory cytokines and circulating CD34^+^ cells [86]. Interestingly, the response to Everolimus was independent of the mutation status of JAK2 or MPL [86]. These results demonstrate that inhibition of mTOR by rapalogs can provide symptomatic relief leading to improvement in quality of life.

In addition to the rapalogs, ATP competitive inhibitors of mTOR complex have been developed. Since these inhibitors inihbit both mTORC complexes, they target the upstream mTORC2 complex that activates AKT as well as its downstream substrate mTORC1 complex thereby effectively acting on the pathway at 3 nodes (Fig. 2). The growth factor independent proliferation of 32D cells with mutant KIT was susceptible to inhibition by the mTORC1/2 inhibitor, AZD8055 [33]. Similarly, in another recent pre-clinical study, PP242, a ATP competitive inhibitor of mTOR was shown to inhibit cell cycle progression and colony formation as effectively as RAD001 (everolimus) in erythropoietin-independent cell lines and progenitor cells from PMF and PV patients with JAK2^V617F^ mutation. It is worth noting that PP242 was also effective against RAD001 resistant JAK2^V617F^ human cell lines and had pro-apoptotic activity in contrast to the cytostatic activity of RAD001 [87]. These differences can be attributed to the ability of PP242 to inhibit both TORC1/2 while RAD001 inhibits only TORC1. 4E-BP1 has rapamycin resistant phosphorylation at Thr^37/46^ that is possibly mediated through serine/threonine kinase Pim2 and maintains cap dependent translation [88]. Therefore, catalytic site inhibitors of mTOR are more likely to be successful as therapeutic drugs. However, while the inhibitors effectively block mTOR activity, they also block the feedback inhibition leading to increase in AKT Thr^308^ phosphorylation by PI3K/PDK1 hyper activation [89]. Taken together the data from pre-clinical and clinical trials supports a pivotal role for mTOR in pathogenesis of MPNs. Further, taking into consideration the moderate success observed in patients that had already developed resistance, inclusion of mTOR inhibitors in the first line therapy may prove to be more beneficial.

#### Inhibitors of AKT/PKB

AKT, a serine threonine kinase with three isoforms encoded by different genes, is another potential target in the PI3K signaling pathway. AKT is recruited to the cell membrane in response to the lipid second messenger, PIP3 through its pleckstrin homology domain where it is phosphorylated by phosphoinositide-dependent protein kinase 1 (PDK1) and mTORC2 at Th^308^ and S^473^ which in turn regulates TSC2/ mTORC1, GSK3β, MDM2 and Bcl2 family proteins. Inhibitors of the AKT kinases in the clinic include ATP-competitive molecules, allosteric inhibitors, lipid mimetics and antisense nucleotide (Table 2). Some of these inhibitors in the pre clinical studies using in vitro and mouse models of MPN have shown promise. An allosteric, non-ATP competitive and orally bioavailable AKT inhibitor, MK-2206, has been established as safe and tolerable in Phase I clinical trials in patients with solid tumors. MK-2206 inhibited AKT phosphorylation in MPN cell lines with JAK2^V617F^ mutation as well as in CD34^+^ peripheral blood cells. This was associated with reduction in phosphorylation of pro-apoptotic protein, BAD and induction of cell death [90]. MK-2206 was also efficacious in a mouse model of myelofibrosis induced by transplanting MPL^W515L^ expressing cells. Treatment with MK-2206 was associated with reduction in CD41^+^ cells and AKT phosphorylation and in the bone marrow, significant reduction in peripheral WBC and megakaryocytes in liver, spleen and bone marrow with no overt hematological toxicity [90]. Interestingly activation of AKT in AML patients contributes to acquisition of resistance to Gemtuzumab ozogamicin(GO), an anti-CD33 antibody conjugated to a calicheamicin-γ(1) derivative and MK-2206 can sensitize these cells in vitro [91]. These results provide further evidence of activation of the PI3K pathway in MPNs with mutations in cytokine/JAK2 pathway and are encouraging for specific targeting of AKT during the chronic and leukemic phases. However, the benefits in the clinic are yet to be realized.

Two other inhibitors of AKT activation, UCN-01, a staurosproine derivative that blocks PDK1 mediated phosphorylation of AKT [92] and Perifosine an orally available alkylphospholipid that prevents recruitment of AKT to membrane for activation [93], were evaluated in combination in a phase I trial in AML and MDS patients. Though the combination was well tolerated and phosphorylation of ribosomal protein S6 was reduced in the blast samples, the combination was not effective in inhibiting AKT phosphorylation [94]. However it is worth nothing that a demonstrable increase in baseline phosphorylation of the AKT target was not present in all the patients. Furthermore, validity of using S6 phosphorylation as biomarker for AKT/mTORC1 inhibition is questionable as phosphorylation of 4E-BP1, another mTORC1 target does not correlate with S6 phosphorylation [88]. These results highlight the need for sensitive and predictive biomarkers for assessing the effectiveness of the inhibitors. It is also possible that all patients may not have constitutive activation of AKT and therefore, may not be good candidates for therapeutic regimens based on inhibition of AKT. A more personalized approach based on molecular characteristics of individual patients is more likely to be successful. Additionally, as discussed below, it might be more prudent to combine inhibitors that target different pathways rather than targeting the same molecule.

#### Inhibitors with dual specificity

Development of dual specificity inhibitors is an alternative approach to blocking the activation of PI3K/AKT/mTOR pathway at multiple levels rather than a single node at a time. These take advantage of the high degree of homology in the kinase domains of PI3K and mTOR. The dual specificity inhibitors bind to the active site of both, PI3K and mTOR. BEZ235, a dual specificity inhibitor of the PI3K/ mTOR pathway developed by Novartis inhibited the proliferation of growth factor independent, clonal proerythroblastic murine cells as well as human mast cell line carrying the D816V mutation with cells being arrested in G1 phase [95, 96]. It was interesting to note that despite increasing the drug concentration, complete inhibition could not be achieved. Interestingly, there was no increase in cell death at growth inhibitory concentrations. Cell proliferation and survival may be regulated by parallel pathways and it would be possible to get synergism by a combination that targets these two pathways. Consistent with this notion, strong synergy and complete inhibition of proliferation with induction of cell death was observed when BEZ235 was combined with UO126 that abrogated MEK/ERK signaling or Obatoclax that blocked pro-survival signaling by the Bcl2 family proteins [96]. In contrast, in cells expressing JAK2^V617F^, BCR-ABL1 or BRAF^V600K^, treatment with BEZ235 was able to overcome the resistance to inhibitors conferred by mutation in G protein β 1 subunit and induced apoptosis [97, 98]. In addition to being effective against cell lines carrying the JAK2 mutation and primary MF cells, BEZ235 also demonstrated synergism with JAK2 inhibitors, TG101209 and SAR302503 [97]. XL147, a pan PI3K inhibitor is in phase I trial in patients with advanced solid cancers. It was interesting to note that as expected, XL147 inhibited phosphorylation of the downstream targets of PI3K pathway (AKT, PRAS40, 4EBP1, and S6) and in addition also inhibited the MEK/ ERK pathway [99]. Compensatory activation of the MEK/ERK pathway plays an important role in development of resistance to PI3K inhibitors. Therefore, co-inhibition of both the pathways by the same molecule would eliminate signaling by both PI3K pathway and the drug resistance inducing MEK/ERK pathway. In addition to the dual specificity inhibitors that target PI3K and mTOR a unique dual specificity inhibitor, CUDC-907 has entered the clinic (Table 2) [100]. CUDC-907 combines the inhibitory activity for class I and IIb histone deacetylase (HDAC) enzymes with inhibition of PI3K α, β and δ isoforms [101]. Acetylation of proteins and their deacetylation by HDACs regulate the activity of several cellular proteins in addition to that of the histones. One such protein is HSP90, a chaperone protein. Increase in acetylation in presence of HDACi leads to increase in proteasomal degradation of mutant oncoproteins such as JAK2, KIT and AKT [102]. The loss of mutated oncoproteins will therefore, lead to elimination of the pathogenic clones. Thus CUDC-907 simultaneously targets both the driving oncoprotein and the aberrant PI3K signaling. Since many of the MPNs also carry mutations in proteins that deregulate the epigenome, combining the epigenetic modifying activity with inhibition of signaling holds promise. The complete data is not yet available but the early results indicate that CUDC also targets the microenvironment due to its anti-angiogenic activity [101]. Earlier the emphasis had been on the specificity of the chemical inhibitors. However, the current trends and results from the clinic show emergence of drug resistance due to hyper activation of compensatory pathways when one single pathway is targeted. With the emergence of the concept of ‘signaling networks’ rather than ‘linear pathways’, molecules that target different nodes in the network would possibly be more effective in shutting down a ‘pathway’ rather than targeting a single node.

#### Combination therapy

Constitutional toxicities, sub-optimal efficacy at lower doses and frequent development of resistance due to activation of compensatory survival pathways have limited the success with single drug based therapies. In addition the complex molecular heterogeneity usually found in MPNs and a very variable evolutionary trajectory in patients makes it a challenge to target with a single drug. Though MPL and JAK2 mutations would activate the same downstream signaling pathway in PMF patients but have different prognosis in terms of median survival and leukemic transformation [29]. This is likely to be influenced by other mutations and cytogenetic abnormalities. Over the course of the disease distinct clones may evolve with different combinations of mutations. Coexistence of subclones with different combinations of genetic aberrations and drug sensitivities makes it harder to target with a single molecule. Patients with CALR mutation may respond to JAK2 inhibitor but when it is present in combination with mutation in SETBP, the cells are resistant to the same treatment [46]. More such studies are needed to clarify how additional mutations contribute to the sensitivity of the cells to these drugs but it is reasonably clear that no single drug is likely to be successful for any of these MPNs. The treatment strategies will have to be more personalized to account the combination of mutations and presence of sub clones. Co-targeting different pathways either sequentially or concomitantly is likely to be more effective strategy in such a scenario. In order to identify the pathways that work synergistically with the constitutionally active JAK pathway, Choong et al. [103] used a panel of small molecule inhibitors directed against CDK, MEK/ERK, JNK, p38 MAPK, Raf, PI3K and mTOR in combination with JAK2 inhibitor ruxolitinib. They found significant synergism with mTOR and MEK/ERK inhibitors while inhibitors of other signaling pathways had little or no effect. Interestingly, most potent synergistic effect was observed with pan PI3K inhibitors (ZSTK474 and GDC0941) and PI3K/mTOR dual inhibitor NVP-BEZ235. When used in combination they inhibited phosphorylation of STAT3/5 and p70S6 Kinase/S6 ribosomal protein, the targets of the JAK and PI3K pathway [103]. BEZ235 also demonstrated synergism with other JAK2 inhibitors, TG101209 and SAR302503 [97] as well as UO126, inhibitor of MEK/ERK signaling and Obatoclax, inhibitor of Bcl2 family proteins [96]. While the combination of PI3K inhibitor with JAK2 inhibitor did not prolong the survival of mice transplanted with JAK2^V617F^ cells, there was 70 % and 60 % reduction in spleen weight as compared to the vehicle treated controls and single drug treated groups, respectively [103]. A relief from the symptoms of the disease can improve the quality of life. Similar synergistic effects were observed using RAD001, allosteric or PP242, ATP competitive inhibitors of mTOR in combination with JAK2 inhibitors ruxolitinib or AZD1480 in proliferation and clonogenic assays using mouse and human cells carrying JAK2^V617F^ mutation [87]. Interestingly, INCB040093 another p110 δ inhibitor in phase I trial (Table 2), was ineffective as a single agent in DLBCL cells with activated JAK/STAT signaling pathway but the cells could be sensitized by a combination treatment with JAK1 inhibitor [104]. Upon combining PI3K inhibitor with JAK inhibitor there was little effect on AKT phosphorylation [103] suggesting that alternative pathways can activate AKT and their concomitant suppression may enhance the therapeutic potential. Consistent with this notion, highly synergistic inhibition of proliferation was observed when AKT inhibitor, MK-2206 was combined with ruxolitinib in human cell line with JAK2^V617F^ mutation [90]. In addition to targeting the proliferating myeloid clones, a combination of PI3K inhibitor PX-866 with MEK inhibitor also attenuated lung fibrosis in preclinical models [73] and may benefit MF patients. These results look promising and provide a strong rationale for combining PI3K inhibitor with JAK2 inhibitors in the management of MPNs. However, these pre-clinical studies are based on cells carrying a single mutation (JAK2) and may not accurately represent the situation in vivo with multiple mutations in the same clone. Development of in vitro assays with patient samples can overcome this limitation. Development of mice models with complex mutations that more closely mimic the patient mutation profiles are likely to be more informative in pre-clinical evaluations for drug combinations.

## Conclusions

Discovery of the genetic mutations and understanding of the signaling pathways involved the pathogenesis of MPNs has led to the rational development of small molecule inhibitors that target the signaling intermediaries. Identification of novel mutations in CALR in ET and PMF patients with non-mutated JAK2 and MPL is another important step in this field but the mechanistic details of how CALR contributes to the development of MPN remains to be elucidated. Additionally, the molecular drivers of aberrant signaling in Ph-ve MPN patients that do not have mutations in JAK2, MPL, KIT, CALR and CSF3R need to be identified. Though the role of deregulated cytokine signaling has become clearer, the functional significance of mutations in epigenetic regulators that are frequently associated with MPNs is largely unknown. However it is clear that PI3K pathway plays a pivotal role in the pathogenesis of many of these Ph-ve MPNs and holds promise for future informed therapeutic decisions. Though we are still far from finding a cure, the promising results from preclinical and clinical testing indicate that co-targeting of the PI3K pathway along with the tyrosine kinases and/or epigenetic regulators will significantly improve the response and lead to better disease management. Since a large number of inhibitors are already in safety and efficacy trials for solid tumors and other hematological malignancies, transition from lab to the clinic for MPN will be a rapid process. The major challenge with PI3K inhibitors will involve the management of the feedback inhibitory loops which are also likely to be compromised by these inhibitors. With improved understanding of the regulatory mechanisms and crosstalk between different signaling pathways, it may be possible to overcome the resistance due to activation of compensatory pathways. Future progress will involve a more personalized approach and therapeutic strategies based on the presence of specific genetic and molecular abnormalities in MPN patients. Better in vitro and in vivo models that mimic the complexity in the mutations observed in the patients would more accurately predict the efficacy of drugs. Improvements in patient selection and rational combinations targeting multiple pathways that synergize with PI3K signaling module is more likely to be successful in realizing the full potential of this promising class of inhibitors.

## Abbreviations

MPN: myeloproliferative neoplasm
BCR: B cell receptor
ABL1: Abelson tyrosine protein kinase 1
Ph: Philadelphia Chromosome
CML: Chronic myeloid leukemia
JAK2: Janus kinase 2
PI3K: Phosphatidylinositol 3 kinase
PV: Polycythemia vera
ET: Essential thrombocythemia
PMF: Primary myelofibrosis
WHO: World Health Organization
PDGFR: Platelet derived growth factor receptor
FGFR: Fibroblast growth factor receptor
CNL: Chronic neutrophilic leukemia
CEL-NOS: Chronic eosinophilic leukemia not otherwise specified
FDA: Food and drug administration
IL-3: Interleukin 3
IFN-γ R2: Interferon gamma receptor 2
MPL: Thrombopoietin receptor (TPO-R)
CALR: Calreticulin
AML: Acute myeloid leukemia
MDS: myelodysplastic syndrome
CMML: Chronic myelomonocytic leukemia
STAT5: Signal transducer and activator of transcription 5
KIT: Stem cell factor receptor
CSF3R: Colony-stimulating factor 3 receptor
SETBP1: SET binding protein 1
TKI: Tyrosine kinase inhibitor
AKT: Protein kinase B
mTOR: Mammalian target of rapamycin
PTEN: Phosphatase and tensin homolog
SHIP: SH2-containing inositol phosphatase
TSC: Tuberous sclerosis proteins
CLL: Chronic Lymphocytic Leukemia
BTK: Bruton’s tyrosine kinase
ATP: Adenosine triphosphate
PDK1: Phosphoinositide-dependent protein kinase 1
WBC: White blood cells
GSK3: Glycogen synthase kinase 3
MDM2: Mouse double minute 2
Bcl2: B-cell lymphoma 2
BAD: Bcl-2-associated death promoter protein
MEK: Mitogen-activated protein kinase kinase
ERK: Extracellular signal-regulated kinases
HDACi: Histone deacetylase inhibitor
CDK: Cyclin-dependent kinase
MAPK: Mitogen-activated protein kinase
JNK: c-Jun N-terminal kinase
